# Pre-notification and personalisation of text-messages to retain participants in a smoking cessation pregnancy RCT: an embedded randomised factorial trial

**DOI:** 10.12688/f1000research.51964.1

**Published:** 2021-07-22

**Authors:** Elizabeth Coleman, Rachel Whitemore, Laura Clark, Karen Daykin, Miranda Clark

**Affiliations:** 1 York Trials Unit, Department of Health Sciences, University of York, UK, York, YO10 5DD, UK; 2 Division of Primary Care, Tower Building, University of Nottingham, Nottingham, NG7 2RD, UK

**Keywords:** Randomised Controlled Trial, Embedded Trial, SWAT, Retention, text, notification, personalisation, SMS

## Abstract

Background:

Low response rates in randomised controlled trials can compromise the reliability of the results, so ways to boost retention are often implemented. Although there is evidence to suggest that sending a text message to participants increases retention, there is little evidence around the timing or personalisation of these messages.

Methods:

A two-by-two factorial SWAT (study within a trial) was embedded within the MiQuit-3 trial, looking at smoking cessation within pregnant smokers. Participants who reached their 36-week gestational follow-up were randomised to receive a personalised or non-personalised text message, either one week or one day prior to the telephone follow-up. Primary outcomes were completion rate of questionnaire via telephone. Secondary outcomes included: completion rate via any method, time to completion, and number of reminders required.

Results

In total 194 participants were randomised into the SWAT; 50 to personalised early text, 47 to personalised late text, 50 to non-personalised early text, and 47 to non-personalised late text. There was no evidence that timing of the text message (early: one week before; or late: one day before) had an effect on any of the outcomes. There was evidence that a personalised text would result in fewer completions via telephone compared with a non-personalised text (adjusted OR 0.44, 95% CI 0.22–0.87, p=0.02). However, there was no evidence to show that personalisation or not was better for any of the secondary outcomes.

Conclusion

Timing of the text message does not appear to influence the retention of participants. Personalisation of a text message may be detrimental to retention; however, more SWATs should be undertaken in this field.

## Introduction

Randomised controlled trials (RCTs) are the ‘gold standard’ for evaluating healthcare treatments. However, it is well documented that retaining participants can be difficult and low response rates to questionnaires can compromise the reliability and generalisability of the results ^1, 2^. A study within a trial (SWAT) can be used to test interventions to improve retention of participants ^3^.

There is research to support the concept that text messages are effective at improving response rates in trials ^4– 7^. There is insufficient evidence to determine if the timing of text messages improves questionnaire response rates, and limited papers exploring if personalisation (inclusion of the participants name) impacts response rate ^8– 11^. This SWAT aims to evaluate the effectiveness of the timing and personalisation of text messages within an RCT to add to the evidence base for both of these interventions. 

## Methods

### Design

This two-by-two factorial study was embedded within the MiQuit-3 RCT. MiQuit-3 (ClinicalTrials.gov NCT03231553) is an RCT evaluating the effectiveness of a text-message, smoking cessation self-help support programme for pregnant smokers (MiQuit), and the protocol has been published previously ^12^. This SWAT was embedded at the 36-week gestational time point. The approval for this SWAT and the MiQuit-3 trial was granted by East Midlands–Nottingham 1 Research Ethics Committee (NRES reference 13/EM/0427 and 17/EM/0327). As the SWAT was considered low risk, informed consent was not obtained from participants, and they were unaware of the SWAT. However, as part of the MiQuit-3 trial all participants consented to their anonymised data being used for further research, and being published. The SWATs are also registered with the Northern Ireland Hub for Trial Methodology Research SWAT Repository (SWATs 35 and 44; both registered December 2015).

### Participants and randomisation

As with all SWATs, the sample size is limited by that of the host trial, and a formal power calculation has not been carried out. The SWAT was implemented mid-way through follow up for the host trial, and all participants that had not yet had their 36-week gestational follow-up were eligible to participate in the SWAT.

Participants in MiQuit-3 were blind to their participation in this SWAT; and were randomised 1:1:1:1 to each of the four groups (see Table 1). The randomisation was undertaken by a statistician independent of the host trial, and of the staff involved in sending the texts. Block randomisation, stratified by host trial allocation, and whether they had completed the previous follow-up; with varying block sizes of 4, 8, 12 and 16.

**Table 1.** Details of the SWAT interventions and combinations.

		SWAT 1 – Personalisation			
		Intervention 1: Personalised	Control 1: Non-personalised
**SWAT 2 – Timing**	**Intervention 2: Early** ** notification**	MiQuit Trial: Hi [name], Thank you for taking part in the MiQuit3 trial. A member of the MiQuit3 team will call next week to complete the final questionnaire. Once completed we will send you a £ 5 or £35 voucher. Whether you have quit smoking or not we would love to speak to you. Thanks, [Researchers name].	MiQuit Trial: Thank you for taking part in the MiQuit3 trial. A member of the MiQuit3 team will call next week to complete the final questionnaire. Once completed we will send you a £ 5 or £35 voucher. Whether you have quit smoking or not we would love to speak to you. Thanks, [Researchers name].
	**Control 2: Late ** **notification**	MiQuit Trial: Hi [name], Thank you for taking part in the MiQuit3 trial. A member of the MiQuit3 team will call tomorrow to complete the final questionnaire. Once completed we will send you a £ 5 or £35 voucher. Whether you have quit smoking or not we would love to speak to you. Thanks, [Researchers name].	MiQuit Trial: Thank you for taking part in the MiQuit3 trial. A member of the MiQuit3 team will call tomorrow to complete the final questionnaire. Once completed we will send you a £ 5 or £35 voucher. Whether you have quit smoking or not we would love to speak to you. Thanks, [Researchers name].

### Interventions

This SWAT explored two different interventions; personalisation and timing of text messages (early; one week before follow-up, or late; one day before follow-up). Details of the text sent to participants can be found in Table one. A £5 voucher was given to all participants who completed a follow-up, additionally those who provided a saliva sample were given another £30 (£35 total).

### Outcomes

The primary outcome was completion rate; defined as the proportion of the questionnaires completed over the telephone within the follow-up window (14 days).

### Secondary outcome measures

The secondary outcome measures included:

- Completion rate where the questionnaire was completed by any method within the follow-up window (14 days)
- Time to response, defined as the number of days between the due date of the 36-week gestation follow-up and the date the questionnaire was recorded as complete
- Number of attempts to contact required before the questionnaire was complete, or the maximum number of calls is reached.

### Statistical analysis

The data were analysed in Stata v.15 (RRID:SCR_012763) on an intention-to-treat (ITT) basis, using two-sided tests at the 2.5% level, as this is a factorial design the Bonferroni correction was applied to allow for multiple testing ^13, 14^. Participants were excluded from the analysis if they had withdrawn prior to the time point.

The primary outcome and completion for all methods were compared using a logistic regression model. Time to response (days between questionnaire due and complete) was analysed using a Cox Proportional Hazards regression, those who compared the questionnaire early had their time set to 0.1, those did not complete were censored at either last contact date or 120 days if not contacted, and those who withdrew in the course of the SWAT were set to their withdrawal date. The assumptions for this model were assessed using Schoenfeld residuals ^15^. The number of attempts to contact was analysed using a negative binomial regression model, due to evidence of overdispersion. All models were adjusted for host trial allocation, whether the participant had completed the previous follow-up, age, and both SWAT intervention allocations. All models were repeated with the inclusion of an interaction term to explore any possible interactions between the two SWAT interventions; with a significance level of 5%.

Stata is proprietary software: a freely available alternative software that could be used to undertake this analysis is RStudio (RRID:SCR_000432 ) ^16^.

## Results

**Figure 1.**
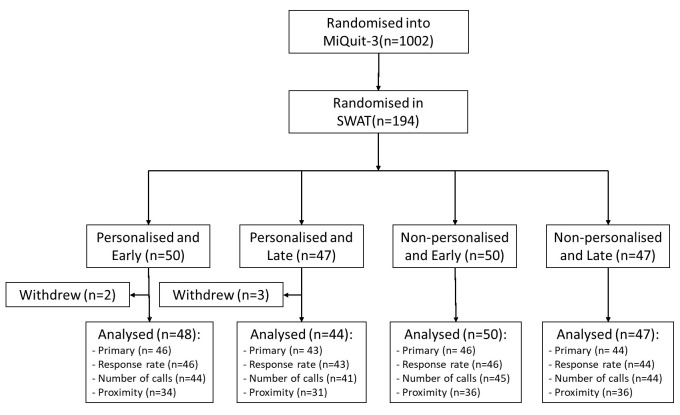
Flow of participants through the SWAT.

In total, 194 participants were randomised into the SWAT; 50 received the personalised text and early notification, 47 received the personalised text and late notification, 50 received the non-personalised text and early notification, and 47 received the non-personalised text and late notification ^17^. Five participants withdrew prior to the implementation of the SWAT and are not included in the analysis. Additional participants were excluded from the analysis, where the covariates required for the model were not provided. Three participants were not contacted due to difficulties/adverse events associated with their pregnancy but are still included in the analysis under ITT principles. The flow of participants can be seen in Figure 1. Baseline characteristics by SWAT arm and overall, can be found in Table 2.

**Table 2.** Baseline characteristics for participants by SWAT allocation.

	Personalised & Early (n=48)	Personalised & Late (n=44)	Non-personalised & Early (n=50)	Non-personalised & Late (n=47)	Overall (n=189)
**Age**	N=48	N=44	N=46	N=44	N=182
**Mean (SD)**	25.4 (5.9)	27.9 (5.9)	27.1 (5.3)	27.2 (6.7)	26.9 (6.0)
**Median (min., max.)**	24 (17, 41)	27 (17, 41)	26 (16, 39)	28 (17, 41)	26 (16, 41)
**Ethnicity: n(%)**					
**Caucasian**	43 (89.6)	42 (95.5)	43 (86.0)	40 (85.1)	168 (88.9)
**Non-Caucasian**	3 (6.3)	1 (2.3)	2 (4.0)	4 (8.5)	10 (5.3)
**Missing**	2 (4.2)	1 (2.3)	5 (10.0)	3 (6.4)	11 (5.8)
**Host trial allocation: n(%)**					
**Intervention**	23 (47.9)	19 (43.2)	24 (48.0)	22 (46.8)	88 (46.6)
**Usual Care**	23 (47.9)	24 (54.6)	22 (44.0)	22 (46.8)	91 (48.2)
**Missing**	2 (4.2)	1 (2.3)	4 (8.0)	3 (6.4)	10 (5.3)
**Completed Previous** **Follow-up: n(%)**					
**Yes**	38 (79.2)	37 (84.1)	36 (72.0)	35 (74.5)	146 (77.3)
**No**	8 (16.7)	7 (15.9)	10 (20.0)	9 (19.2)	34 (18.0)
**Missing**	2 (4.2)	0 (0.0)	4 (8.0)	3 (6.4)	9 (4.8)

### Primary outcome

The overall completion rate by telephone was 66.1% (125/189) within 14 days of the due date. There were similar completion rates of the questionnaire *via* telephone within three groups; 50.0% for personalised early (24/48), 52.3% (23/44) for personalised late, and 58.0% (29/50) of non-personalised early and was slightly higher in the non-personalised late group, 66.0% (31/47).

There was no evidence for a difference in completion rate *via* telephone for the timing of the text message; adjusted odds ratio (OR) 0.86 (95% CI 0.44–1.67, p=0.65). There was evidence to suggest a difference in completion rate *via* telephone adjusted OR 0.44 (0.22–0.87, p=0.02) which implies those who received the non-personalised text were more likely to complete the questionnaire when completing *via* the telephone. Full details can be found in Table 3.

**Table 3.** Primary analysis results.

Primary Outcome	Group	Statistic *	95% Confidence Interval	p-value
**Response rate for** ** all methods**	Personalised vs. non-personalised	OR = 0.44	0.22 to 0.87	0.02
	Early *versus* Late	OR = 0.86	0.44 to 1.67	0.65
	Host trial allocation (Intervention *versus* Control)	OR = 0.63	0.32 to 1.22	0.17
	Completed previous follow-up (Yes *versus* No)	OR = 9.90	3.87 to 25.35	>0.001
	Age (years)	OR = 1.02	0.96 to 1.07	0.60

* OR = Odds Ratio

Secondary outcomes:

Full details for all secondary outcomes can be found in Table 4.

**Table 4.** Results for the secondary analyses.

Secondary Outcome	Group	Statistic *	95% Confidence Interval	p-value
**Response rate for** ** all methods**	Personalised vs. non-personalised	OR = 0.61	0.30 to 1.24	0.17
	Early *versus* Late	OR = 1.06	0.52 to 2.15	0.87
	Host trial allocation (Intervention *versus* Control)	OR = 0.79	0.39 to 1.60	0.51
	Completed previous follow-up (Yes *versus* No)	OR = 8.45	3.60 to 19.86	>0.001
	Age (years)	OR = 1.05	0.99 to 1.11	0.12
**Number of ** **attempted to ** **contact required**	Personalised vs. non-personalised	IRR = 1.14	0.92 to 1.41	0.23
	Early *versus* Late	IRR = 1.08	0.88 to 1.33	0.45
	Host trial allocation (Intervention *versus* Control)	IRR = 1.11	0.90 to 1.37	0.33
	Completed previous follow-up (Yes *versus* No)	IRR = 0.64	0.50 to 0.82	>0.001
	Age (years)	IRR = 1.00	0.98 to 1.02	0.79
**Time to response**	Personalised vs. non-personalised	HR = 0.76	0.54 to 1.07	0.12
	Early *versus* Late	HR = 1.00	0.71 to 1.40	0.99
	Host trial allocation (Intervention *versus* Control)	HR = 0.87	0.62 to 1.21	0.40
	Completed previous follow-up (Yes *versus* No)	HR = 3.42	1.95 to 5.99	>0.001
	Age (years)	HR = 1.01	0.98 to 1.04	0.51

* OR = Odds Ratio, IRR = Incidence Rate Ratio, HR = Hazards Ratio

***Response rates for all methods.*** There were similar completion rates of the questionnaire within each of the four groups; 64.6% for personalised early (31/48), 63.6% (28/44) for personalised late, 66.0% for early (33/50) and 70.2% (33/47) of non-personalised.

There is some, non statistically significant, evidence to suggest that there may be a difference in response rate for personalised *versus* non-personalised text reminders; adjusted OR 0.61 (95% CI 0.30–1.24, p=0.17), in favour of the non-personalised text messages. However, there was no evidence to suggest there was a difference in response rates in participants who received an early or late text message reminder; adjusted OR 1.06 (95% CI 0.52–2.15, p=0.87).

***Number of attempts to contact required.*** The average number of calls required was 3.0 for all participants, with the average similar for each group (3.3 for both personalised early, 3.2 for personalised late, 3.1 for non-personalised early and 2.7 for non-personalised late). The maximum number of calls was reached for 55 of the 174 participants (31.3%) and was similar across three groups (38.6% for personalised and early, 31.7% for personalised and late, 31.1% for non-personalised early) and slightly lower in the non-personalised late group, 25%.

There was no evidence of a difference in number of contacts required between those who received an early text or a late text (p=0.45). There is also no evidence to suggest a difference between those who received a personalised or non-personalised text (p=0.23); adjusted incidence rate ratio (IRR)=1.14.

***Time to respond.*** The average time to respond was 6.2 days (ranging from 5 days early to 103 days late). This was similar between those who received a personalised text (8.2 days for early *versus* 7.1 days for late) and those who received the non-personalised text (4.9 days for early *versus* 4.7 days for late), but there is a slight difference between those who received personalised or non-personalised texts.

There was no evidence of a difference in time taken to respond between those who received the text early or late (p=0.99) or those who received a personalised or non-personalised text (p=0.12); suggesting that neither timing nor personalisation of the text message reminder affect the time taken to complete the questionnaire. The assumptions for the model held when examined using Schoenfeld residuals (p=0.66).

***Interaction terms.*** All of the models were re-run with the inclusion of any interaction term between the two SWAT allocations. There was no evidence of an interaction for the completion rate, both by phone only (p=0.57) and all methods (p=0.54). There was also no evidence of an interaction for the number of contacts required (p=0.69), or the time to respond (p=0.88).

There were 1002 participants who were randomised into the MiQuit-3 trial. Of the 777 who were not included in the SWAT, and were due a 36-week follow-up, 499 completed the questionnaire (64.2%). This is similar to the completion rate for the participants in the SWAT (overall 66.1%).

## Discussion

This factorial SWAT showed that the timing of the text message reminder had no effect on the response rate, the time to response, or the number of attempted to contact required; these results mirror what Partha *et al.* reported in their work ^8^. It also showed that personalised texts have no effect on response time, or number of attempts required. It did show that there was some evidence that sending a non-personalised text message reminder would have a larger increase in response than sending personalised text messages did. Cochrane *et al*. found no statistically significant difference in their study, but results favoured the non-personalised text ^11^. As our work was conducted in a female-only population, who were between 17 and 41 years of age, the results here are only directly related to this population. Equally, as the SWAT was not powered to detect a difference, more SWATs should be undertaken in this area to allow the results to be combined in a pooled analysis to determine the true effect of the interventions, consider the effects on a wider population, and overall effectiveness.

## Data availability

### Underlying data

Figshare: Underlying data for ‘Pre-notification and personalisation of text-messages to retain participants in a smoking cessation pregnancy RCT: an embedded randomised factorial trial’. https://doi.org/10.6084/m9.figshare.14224319.v1 ^17^

Data are available under the terms of the Creative Commons Attribution 4.0 International license (CC-BY 4.0).

### Reporting guidelines

Figshare: CONSORT checklist for ‘Pre-notification and personalisation of text-messages to retain participants in a smoking cessation pregnancy RCT: an embedded randomised factorial trial’. https://doi.org/10.6084/m9.figshare.14229647.v1 ^18^
