# Pre-notification and personalisation of text messages to increase questionnaire completion in a smoking cessation pregnancy RCT: an embedded randomised factorial trial

**DOI:** 10.12688/f1000research.51964.2

**Published:** 2021-09-30

**Authors:** Elizabeth Coleman, Rachel Whitemore, Laura Clark, Karen Daykin, Miranda Clark

**Affiliations:** 1 York Trials Unit, Department of Health Sciences, University of York, UK, York, YO10 5DD, UK; 2 Division of Primary Care, Tower Building, University of Nottingham, Nottingham, NG7 2RD, UK

**Keywords:** Randomised Controlled Trial, Embedded Trial, SWAT, Retention, text, notification, personalisation, SMS

## Abstract

Background:

Low completion rates of questionnaires in randomised controlled trials can compromise the reliability of the results, so ways to boost questionnaire completion are often implemented. Although there is evidence to suggest that sending a text message to participants increases completion, there is little evidence around the timing or personalisation of these text messages.

Methods:

A two-by-two factorial SWAT (study within a trial) was embedded within the MiQuit-3 trial, looking at smoking cessation within pregnant smokers. Participants who reached their 36-week gestational follow-up were randomised to receive a personalised or non-personalised text message, either one week or one day prior to their follow-up. Primary outcomes were completion rate of questionnaire via telephone. Secondary outcomes included: completion rate via any method, time to completion, and number of attempts to contact required.

Results

In total 194 participants were randomised into the SWAT to receive a text message that was personalised early(n=50), personalised late (n=47), non-personalised early(n=50), or non-personalised late(n=47). There was no evidence that timing of the text message (early: one week before; or late: one day before) had an effect on any of the outcomes. There was evidence that a personalised text message would result in fewer completions compared with a non-personalised text message when data was collected only via the telephone(adjusted OR 0.44, 95% CI 0.22–0.87, p=0.02). However, these results were not significant when looking at completion via any method (adjusted OR 0.61, 95% CI 0.30-1.24, p=0.17). There was no evidence to show that personalisation or not was better for any of the secondary outcomes.

Conclusion

Timing of the text message does not appear to influence the completion of questionnaires. Personalisation of a text message may be detrimental to questionnaire completion, if data is only collected via the telephone - however, more SWATs should be undertaken in this field.

## Introduction

Randomised controlled trials (RCTs) are the ‘gold standard’ for evaluating healthcare treatments. However, it is well documented that retaining participants can be difficult and low response rates to questionnaires can compromise the reliability and generalisability of the results ^1, 2^. A study within a trial (SWAT) can be used to test interventions to improve retention of participants, via increasing questionnaire completion ^3^.

There is research to support the concept that text messages are effective at improving questionnaire completion rates in trials ^4– 7^. There is insufficient evidence to determine if the timing of text messages improves questionnaire completion rates, and limited papers exploring if personalisation (inclusion of the participants name) impacts questionnaire completion rates ^8– 11^. This factorial SWAT aims to evaluate the effectiveness of the timing and personalisation of text messages within an RCT to add to the evidence base for both of these interventions.

## Methods

### Design

This two-by-two factorial study was embedded within the MiQuit-3 RCT. MiQuit-3 (ClinicalTrials.gov NCT03231553) is an RCT evaluating the effectiveness of a text-message, smoking cessation self-help support programme for pregnant smokers (MiQuit), and the protocol has been published previously ^12^. This factorial SWAT was embedded at the 36-week gestational time point. The approval for this factorial SWAT and the MiQuit-3 trial was granted by East Midlands–Nottingham 1 Research Ethics Committee (NRES reference 13/EM/0427 and 17/EM/0327). As the SWAT was considered low risk, informed consent was not obtained from participants, and they were unaware of the SWAT. However, as part of the MiQuit-3 trial all participants consented to their anonymised data being used for further research and being published. The SWATs that form the factorial design are also registered with the Northern Ireland Hub for Trial Methodology Research SWAT Repository (SWATs 35 and 44; both registered December 2015).

### Participants and randomisation

As with all SWATs, the sample size is limited by that of the host trial, and a formal power calculation has not been conducted. In total 1002 participants were randomised to the MiQuit-3 trial. As this SWAT was implemented mid-way through follow up for the host trial, all participants that had not yet had their 36-week gestational follow-up, approximately 200, were eligible to participate in the SWAT, and any that had already passed this follow-up time point were unable to be included in this SWAT.

Participants in MiQuit-3 were unaware of their participation in this SWAT, however, they could not be blinded to the contents or timing of the text message. Participants were randomised 1:1:1:1 to each of the four groups (see Table 1). The randomisation was undertaken by a statistician independent of the host trial, and of the staff involved in sending the text messages. Block randomisation was used with varying block sizes of 4, 8, 12 and 16, which was stratified by host trial allocation, and whether they had completed the previous follow-up or not. The randomisation sequence was generated in Stata v.15 (RRID:SCR_012763) and implemented using a remote computer system, independent of the researchers.

**Table 1.** Details of the SWAT interventions and combinations.

		SWAT 1 – Personalisation			
		Intervention 1: Personalised	Control 1: Non-personalised
**SWAT 2 – Timing**	**Intervention 2: Early** ** notification**	MiQuit Trial: Hi [name], Thank you for taking part in the MiQuit3 trial. A member of the MiQuit3 team will call next week to complete the final questionnaire. Once completed we will send you a £ 5 or £35 voucher. Whether you have quit smoking or not we would love to speak to you. Thanks, [Researchers name].	MiQuit Trial: Thank you for taking part in the MiQuit3 trial. A member of the MiQuit3 team will call next week to complete the final questionnaire. Once completed we will send you a £ 5 or £35 voucher. Whether you have quit smoking or not we would love to speak to you. Thanks, [Researchers name].
	**Control 2: Late ** **notification**	MiQuit Trial: Hi [name], Thank you for taking part in the MiQuit3 trial. A member of the MiQuit3 team will call tomorrow to complete the final questionnaire. Once completed we will send you a £ 5 or £35 voucher. Whether you have quit smoking or not we would love to speak to you. Thanks, [Researchers name].	MiQuit Trial: Thank you for taking part in the MiQuit3 trial. A member of the MiQuit3 team will call tomorrow to complete the final questionnaire. Once completed we will send you a £ 5 or £35 voucher. Whether you have quit smoking or not we would love to speak to you. Thanks, [Researchers name].

### Interventions

This SWAT explored two different interventions; personalisation and timing of text messages (early; one week before follow-up, or late; one day before follow-up). Details of the text message sent to participants can be found in Table one. As detailed in the MiQuit-3 protocol ^12^, all participants were given a £5 voucher if they completed the 36-week follow-up. Those who provided a saliva sample to validate their smoking status were given an additional £30 voucher. This amount is more than is stated in the published protocol, but this change was made prior to the implementation of this SWAT, and as such all participants involved in this SWAT would have been eligible for this amount. These monetary incentives formed part of the host trial, and where not related to the factorial SWAT being undertaken.

### Outcomes

The primary outcome was completion rate; defined as the proportion of the questionnaires completed over the telephone within the follow-up window (14 days).

### Secondary outcome measures

The secondary outcome measures included:

- Completion rate where the questionnaire was completed by any method (postal, telephone, email/web, or text message) within the follow-up window (14 days)
- Time to completion, defined as the number of days between the due date of the 36-week gestation follow-up and the date the questionnaire was recorded as complete
- Number of attempts to contact required before the questionnaire was complete, or the maximum number of attempts, six, is reached.

Both time to completion and number of attempts to contact were not restricted by method of data collection, and thus included participants who completed (or were being contacted) via any method.

### Statistical analysis

The data were analysed in Stata v.15 (RRID:SCR_012763) on an intention-to-treat (ITT) basis, using two-sided tests at the 2.5% level. As this is a factorial design the Bonferroni correction was applied to allow for multiple testing ^13, 14^. Participants were excluded from the analysis if they had withdrawn prior to the time point.

The primary outcome and completion for all methods were compared using a logistic regression model. Time to completion (days between questionnaire due and complete) was analysed using a Cox Proportional Hazards regression. Participants who completed the questionnaire early had their time set to 0.1, those who did not complete it were censored at either last contact date or 120 days if not contacted, and those who withdrew in the course of the SWAT were set to their withdrawal date. The assumptions for this model were assessed using Schoenfeld residuals ^15^. The number of attempts to contact was analysed using a negative binomial regression model, due to evidence of overdispersion. All models were adjusted for host trial allocation, whether the participant had completed the previous follow-up, age, and both SWAT intervention allocations separately. All models were repeated with the inclusion of an interaction term to explore any possible interactions between the two SWAT interventions. This was done using two-sided tests at a significance level of 5%.

Stata is proprietary software: a freely available alternative software that could be used to undertake this analysis is RStudio (RRID:SCR_000432) ^16^.

## Results

**Figure 1.**
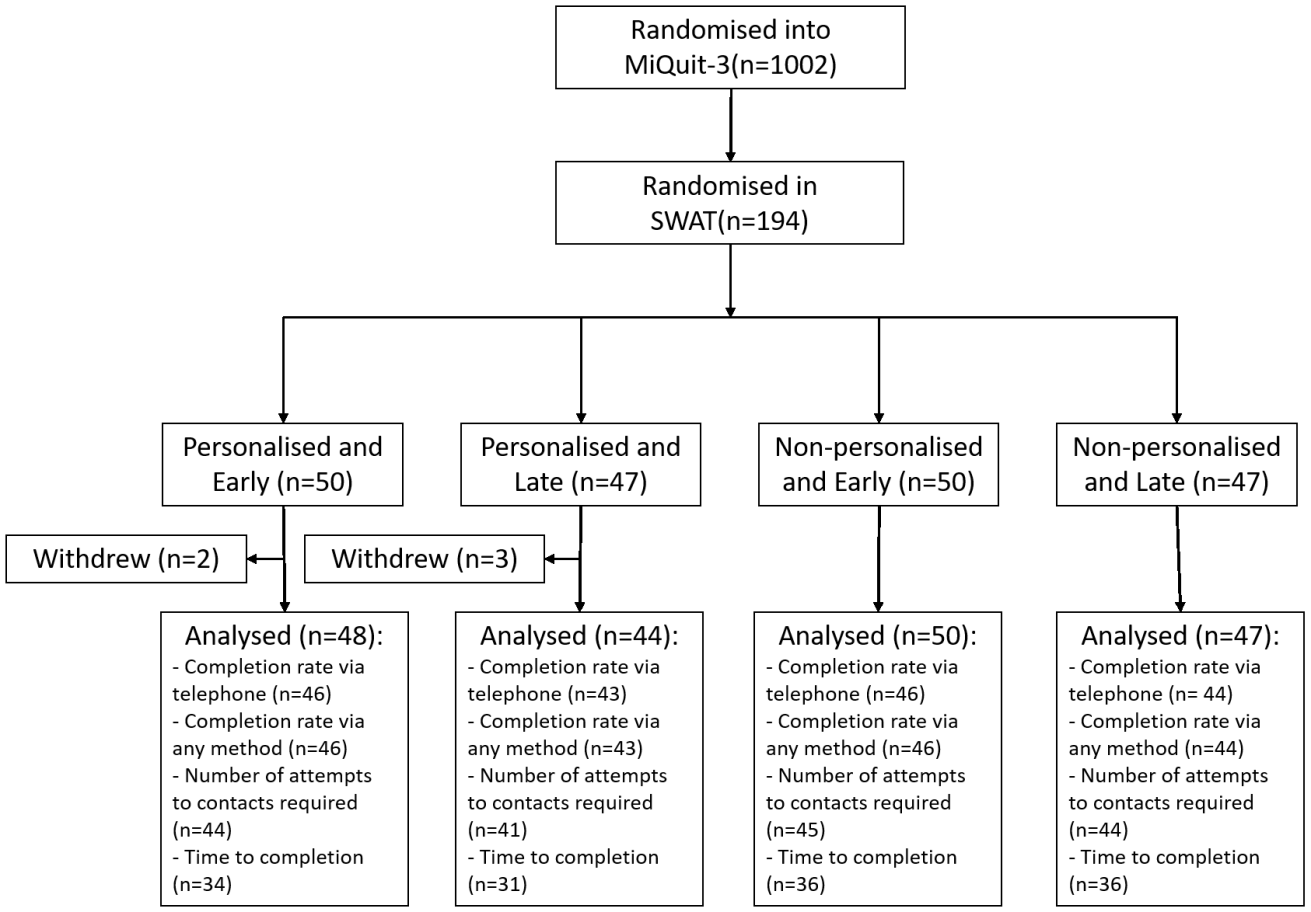
Flow of participants through the SWAT.

In total, 194 participants were randomised into the SWAT; 50 received the personalised text message and early notification, 47 received the personalised text message and late notification, 50 received the non-personalised text message and early notification, and 47 received the non-personalised text message and late notification ^17^. Five participants withdrew prior to the implementation of the SWAT and are not included in the analysis. Participants were only included in a model if all relevant covariates for that model were present. The number included in each of the analysis, by arm, is shown in the flow diagram – Figure 1. Three participants were not contacted due to difficulties/adverse events associated with their pregnancy, but are still included in the analysis under ITT principles. The flow of participants can be seen in Figure 1. Baseline characteristics by SWAT arm and overall, can be found in Table 2.

**Table 2.** Baseline characteristics for participants by SWAT allocation.

	Personalised & Early (n=48)	Personalised & Late (n=44)	Non-personalised & Early (n=50)	Non-personalised & Late (n=47)	Overall (n=189)
**Age**	N=48	N=44	N=46	N=44	N=182
**Mean (SD)**	25.4 (5.9)	27.9 (5.9)	27.1 (5.3)	27.2 (6.7)	26.9 (6.0)
**Median (min., max.)**	24 (17, 41)	27 (17, 41)	26 (16, 39)	28 (17, 41)	26 (16, 41)
**Ethnicity: n(%)**					
**Caucasian**	43 (89.6)	42 (95.5)	43 (86.0)	40 (85.1)	168 (88.9)
**Non-Caucasian**	3 (6.3)	1 (2.3)	2 (4.0)	4 (8.5)	10 (5.3)
**Missing**	2 (4.2)	1 (2.3)	5 (10.0)	3 (6.4)	11 (5.8)
**Host trial allocation: n(%)**					
**Intervention**	23 (47.9)	19 (43.2)	24 (48.0)	22 (46.8)	88 (46.6)
**Usual Care**	23 (47.9)	24 (54.6)	22 (44.0)	22 (46.8)	91 (48.2)
**Missing**	2 (4.2)	1 (2.3)	4 (8.0)	3 (6.4)	10 (5.3)
**Completed Previous** **Follow-up: n(%)**					
**Yes**	38 (79.2)	37 (84.1)	36 (72.0)	35 (74.5)	146 (77.3)
**No**	8 (16.7)	7 (15.9)	10 (20.0)	9 (19.2)	34 (18.0)
**Missing**	2 (4.2)	0 (0.0)	4 (8.0)	3 (6.4)	9 (4.8)

### Primary outcome

The main method of data collection for the MiQuit-3 trial was telephone collection. As such the primary outcome explores the completion rates where the data was collected via telephone calls only. The overall completion rate by telephone was 66.1% (125/189) within the follow-up window (14 days). There were similar completion rates of the questionnaire *via* telephone within three groups; 50.0% for personalised early (24/48), 52.3% (23/44) for personalised late, and 58.0% (29/50) of non-personalised early, and was slightly higher in the non-personalised late group, 66.0% (31/47).

There was no evidence for a difference in completion rate for the timing of the text message where data was collected *via* telephone calls; adjusted odds ratio (OR) 0.86 (95% CI 0.44–1.67, p=0.65). There was evidence to suggest a difference in completion rate (adjusted OR 0.44, 95%CI 0.22–0.87, p=0.02) which implies those who received the non-personalised text message were more likely to complete the questionnaire than those who received a personalised text message, when data was collected *via* the telephone. Full details can be found in Table 3.

***Completion rates for all methods.*** Additional methods of data collection were used alongside telephone calls. For completion via any method the data could have been collected via post, telephone, email/web or text message. When looking at any method, there were similar completion rates of the questionnaire within each of the four groups; 64.6% for personalised early (31/48), 63.6% (28/44) for personalised late, 66.0% for early (33/50) and 70.2% (33/47) of non-personalised.

**Table 3.** Primary analysis results.

Primary Outcome	Group	Statistic *	95% Confidence Interval	p-value
**Completion rate** **for telephone only**	Personalised *versus* non-personalised	OR = 0.44	0.22 to 0.87	0.02
	Early *versus* Late	OR = 0.86	0.44 to 1.67	0.65
	Host trial allocation (Intervention *versus* Control)	OR = 0.63	0.32 to 1.22	0.17
	Completed previous follow-up (Yes *versus* No)	OR = 9.90	3.87 to 25.35	>0.001
	Age (years)	OR = 1.02	0.96 to 1.07	0.60

* OR = Odds Ratio

Secondary outcomes:

Full details for all secondary outcomes can be found in Table 4.

**Table 4.** Results for the secondary analyses.

Secondary Outcome	Group	Statistic *	95% Confidence Interval	p-value
**Completion rate** **for all methods**	Personalised *versus* non-personalised	OR = 0.61	0.30 to 1.24	0.17
	Early *versus* Late	OR = 1.06	0.52 to 2.15	0.87
	Host trial allocation (Intervention *versus* Control)	OR = 0.79	0.39 to 1.60	0.51
	Completed previous follow-up (Yes *versus* No)	OR = 8.45	3.60 to 19.86	>0.001
	Age (years)	OR = 1.05	0.99 to 1.11	0.12
**Number of ** **attempts to ** **contact required**	Personalised *versus* non-personalised	IRR = 1.14	0.92 to 1.41	0.23
	Early *versus* Late	IRR = 1.08	0.88 to 1.33	0.45
	Host trial allocation (Intervention *versus* Control)	IRR = 1.11	0.90 to 1.37	0.33
	Completed previous follow-up (Yes *versus* No)	IRR = 0.64	0.50 to 0.82	>0.001
	Age (years)	IRR = 1.00	0.98 to 1.02	0.79
**Time to completion**	Personalised *versus* non-personalised	HR = 0.76	0.54 to 1.07	0.12
	Early *versus* Late	HR = 1.00	0.71 to 1.40	0.99
	Host trial allocation (Intervention *versus* Control)	HR = 0.87	0.62 to 1.21	0.40
	Completed previous follow-up (Yes *versus* No)	HR = 3.42	1.95 to 5.99	>0.001
	Age (years)	HR = 1.01	0.98 to 1.04	0.51

* OR = Odds Ratio, IRR = Incidence Rate Ratio, HR = Hazards Ratio

There is no evidence to suggest that there is a difference in completion rate for personalised *versus* non-personalised text messages; adjusted OR 0.61 (95% CI 0.30–1.24, p=0.17). Additionally, there was no evidence to suggest there was a difference in completion rates in participants who received an early or late text message; adjusted OR 1.06 (95% CI 0.52–2.15, p=0.87).

***Number of attempts to contact required.*** The average number of attempts to contact required was 3.0 for all participants, with the average similar for each group (3.3 for both personalised early, 3.2 for personalised late, 3.1 for non-personalised early and 2.7 for non-personalised late). Researchers attempted to contact a participant a maximum of six times. The maximum number of attempts to contact was reached for 55 of the 174 participants (31.3%) and was similar across three groups (38.6% for personalised and early, 31.7% for personalised and late, 31.1% for non-personalised early) and slightly lower in the non-personalised late group, 25%.

There was no evidence of a difference in number attempts to contacts required between those who received an early text message or a late text message (p=0.45). There is also no evidence to suggest a difference between those who received a personalised or non-personalised text message (p=0.23); adjusted incidence rate ratio (IRR) 1.14.

***Time to completion.*** The average time to completion of the questionnaire was 6.2 days (ranging from 5 days early to 103 days late). The time to completion was similar between those who received an early or late personalised text message text message (8.2 days for early *versus* 7.1 days for late) and was similar for those who received an early or late non-personalised text message (4.9 days for early *versus* 4.7 days for late). However, there was a slight difference in time to completion between those who received personalised or non-personalised text message.

There was no evidence of a difference in time to completion between those who received the text message early or late (p=0.99) or those who received a personalised or non-personalised text message (p=0.12). This suggest that neither timing nor personalisation of the text message reminder affect the time taken to complete the questionnaire. The assumptions for the model held when examined using Schoenfeld residuals (p=0.66).

***Interaction terms.*** All of the models were re-run with the inclusion of any interaction term between the two SWAT allocations. There was no evidence of an interaction for the completion rate, both by phone only (p=0.57) and all methods (p=0.54). There was also no evidence of an interaction for the number of contacts required (p=0.69), or the time to completion (p=0.88).

***Comparison with the whole RCT.*** There were 1002 participants who were randomised into the MiQuit-3 trial. Of the 777 who were not included in the SWAT, and were due a 36-week follow-up, 499 completed the questionnaire (64.2%). This is similar to the completion rate for the participants in the SWAT (overall 66.1%).

## Discussion

This factorial SWAT showed that the timing of the text message reminder had no effect on the questionnaire completion rate, the time to complete, or the number of attempts to contact required; these results mirror what Partha *et al.* reported in their work ^8^. It also showed that personalised text messages have no effect on completion time, or number of attempts to contact required. However, it did show that there was some evidence that sending a non-personalised text message reminder would have a larger increase in response than sending personalised text messages did, but these finding were only significant when exploring telephone data collection. Cochrane *et al*. found no statistically significant difference in their study, but results favoured the non-personalised text messages ^11^. As our work was conducted in a female-only population, who were between 17 and 41 years of age, the results here are only directly related to this population. Equally, as the SWAT was not powered to detect a difference, more SWATs should be undertaken in this area to allow the results to be combined in a pooled analysis to determine the true effect of the interventions and consider the effects on a wider population.

## Data availability

### Underlying data

Figshare: Underlying data for ‘Pre-notification and personalisation of text-messages to retain participants in a smoking cessation pregnancy RCT: an embedded randomised factorial trial’. https://doi.org/10.6084/m9.figshare.14224319.v1 ^17^

Data are available under the terms of the Creative Commons Attribution 4.0 International license (CC-BY 4.0).

### Reporting guidelines

Figshare: CONSORT checklist for ‘Pre-notification and personalisation of text-messages to retain participants in a smoking cessation pregnancy RCT: an embedded randomised factorial trial’. https://doi.org/10.6084/m9.figshare.14229647.v1 ^18^
