# Supplementary material for: Pre-notification and personalisation of text messages to increase questionnaire completion in a smoking cessation pregnancy RCT: an embedded randomised factorial trial
Source: F1000Res. 2021 Sep 30;10:637. Originally published 2021 Jul 22. [Version 2] doi: 10.12688/f1000research.51964.2 (PMC8491148; doi:10.12688/f1000research.51964.2)
Supplement: Peer review history

# Peer review: Reviewer response for version 2

**DOI:** 10.5256/f1000research.77567.r95844

**Published:** 2021-10-04

**Authors:** Frances Shiely

**Affiliations:** 1 Trials Research and Methodologies Unit, HRB Clinical Research Facility, Mercy University Hospital, University College Cork, Cork, Ireland; 2 School of Public Health, University College cork, Cork, Ireland

Thank you to authors for making the suggested changes. I believe these amendments will allow for this SWAT to be replicated. I have no further comments.

Is the work clearly and accurately presented and does it cite the current literature?

Partly

If applicable, is the statistical analysis and its interpretation appropriate?

Partly

Are all the source data underlying the results available to ensure full reproducibility?

Yes

Is the study design appropriate and is the work technically sound?

Yes

Are the conclusions drawn adequately supported by the results?

No

Are sufficient details of methods and analysis provided to allow replication by others?

Partly

Reviewer Expertise:

Epidemiology; Trial Methodology; SWATs

I confirm that I have read this submission and believe that I have an appropriate level of expertise to confirm that it is of an acceptable scientific standard.

# Peer review: Reviewer response for version 1

**DOI:** 10.5256/f1000research.55180.r90184

**Published:** 2021-08-04

**Authors:** Frances Shiely

**Affiliations:** 1 Trials Research and Methodologies Unit, HRB Clinical Research Facility, Mercy University Hospital, University College Cork, Cork, Ireland; 2 School of Public Health, University College cork, Cork, Ireland

**Summary**

 This was a SWAT within the MiQuit-3 trial. The purpose of the SWAT was to establish if the timing or personalisation of text messages increases completion of a questionnaire. Study design was a 2 x 2 factorial SWAT. Participants who reached their 36-week gestational follow-up were randomised to receive a personalised or non-personalised text message, either one week or one day prior to the telephone follow-up. Primary outcome was completion rate of questionnaire via telephone. Secondary outcomes included: completion rate via any method, time to completion, and number of reminders required. The authors concluded that timing of the text message did not appear to influence the retention of participants. The authors concluded that personalisation of a text message may be detrimental to retention; however, more SWATs should be undertaken in this field.

**Major Comments**

 My first comment is on the title, the purpose of the study and the conclusions drawn. The title, correctly refers to the retention of participants in the smoking cessation pregnancy RCT. The purpose of the SWAT though is to evaluate completion of a questionnaire, not retention in the host trial, as claimed in the conclusion in the abstract. I think this is conflated throughout and the authors need to consider this carefully and amend their paper. In fact, the registered SWATs in the NI SWAT repository give the outcomes in both as questionnaire completion. So therefore the conclusions drawn in the abstract and in the discussion are not supported by the data. 

**Abstract**

- "There was evidence that a personalised text would result in fewer completions via telephone compared with a non-personalised text (adjusted OR 0.44, 95% CI 0.22–0.87, p=0.02)". This statement is confusing. The research question is personalised text message versus non-personalised text message. If using via telephone, then it should be also say non-personalised text via telephone.
- Also, use text message, rather than text, throughout.
- "Personalisation of a text message may be detrimental to retention". I don't think your results support a statement this strong. Firstly, this is not a trial with an adequate sample size to make this claim. Secondly, when you included all methods of receiving the questionnaire (I think this is correct interpretation but it is challenging to establish in the current version of the paper as the detail on all methods is unclear) you did not find that personalised or non-personalised texts mattered. At best you can say, personalisation of a text message appears to affect questionnaire completion via telephone.

**Introduction**

- I like the succinct introduction.

**Methods**

- The methods says "The SWATs are also registered with the Northern Ireland Hub for Trial Methodology Research SWAT Repository (SWATs 35 and 44; both registered December 2015). It's a little confusing to the reader. It looks like you're conducting two SWATs. I understand what you have taken both of these SWATS and conducted the two in a 2 x 2 factorial design. However, make this clear to the reader.
- Replace the phrase "carried out" with conducted throughout. I know the phrase carried out is used all the time in literature but it is not correct unless you are describing physically carrying an object.
- I would use the word women rather than participants, given that it is a trial of pregnant women.
- The phrase "all participants that had not yet had their 36-week gestational follow-up were eligible to participate" suggests that some people were more than 36 weeks pregnant. Is this the case? Otherwise you could simplify it and say women who were 36 weeks pregnant. It's important to be clear on the timing of the intervention given that this is one of your outcomes.
- "Participants in MiQuit-3 were blind to their participation in this SWAT". Blinding is different to being unaware. Were they both blinded and unaware?
- "The randomisation was undertaken by a statistician independent of the host trial, and of the staff involved in sending the texts". Explain how the statistician did the randomisation, e.g. computer generated. Also, explain how he communicated that randomisation to the researcher assigning the women to each group.
- "Block randomisation, stratified by host trial allocation, and whether they had completed the previous follow-up; with varying block sizes of 4, 8, 12 and 16". This is not a sentence.
- "A £5 voucher was given to all participants who completed a follow-up…". Was this part of the host trial or the SWAT?
- "…additionally those who provided a saliva sample were given another £30 (£35 total)." Where does the saliva sample come into it? Is this part of the host trial? Explain in the paper.
- How did you decide on how many to include for the SWAT. I accept that a sample size calculation is not required but a line in the paper on why you decided on (it appears to be 200) would be useful.
- In the secondary outcome, explain what you mean by completed by any method.
- "Time to response, defined as the number of days between the due date of the 36-week gestation follow-up and the date the questionnaire was recorded as complete". Are you certain all follow-up calls were made in 24 hours?

**Statistical Analysis**

- "The primary outcome was completion rate; defined as the proportion of the questionnaires completed over the telephone within the follow-up window (14 days)". However, you then go on to say that you used logistic regression to analyse this. Logistic regression is not suitable for four categories. I suspect what you mean is that you compared the completion rates across the two personalised/not personalised and again early/late. I can see you did this from the table. However, you need to articulate that in the text because it is currently confusing. 
- In statistical analysis, a full stop after level and a new sentence for "As this is a factorial..."
- Full stop required in this sentence too. Also, I suspect the word "compared" in this sentence should read completed. - "Time to response (days between questionnaire due and complete) was analysed using a Cox Proportional Hazards regression, those who **compared** the questionnaire early had their time set to 0.1, those did not complete were censored at either last contact date or 120 days if not contacted, and those who withdrew in the course of the SWAT were set to their withdrawal date".
- Again the following is not a sentence, "All models were repeated with the inclusion of an interaction term to explore any possible interactions between the two SWAT interventions; with a significance level of 5%."

**Results**

- "Additional participants were excluded from the analysis, where the covariates required for the model were not provided". What additional participants? Quantify and explain please. Why were the covariates not 'provided'? Explain please.
- "Three participants were not contacted due to difficulties/adverse events associated with their pregnancy but are still included in the analysis under ITT principles". Commas are required to make sense of the sentence.
- In your flow chart, what does primary refer to, and proximity? Add an explanation or use a term that explains a little better. Also in the flow chart, you say response rate but provide the number of participants. This is not a rate.
- In the primary outcome, continue with your phraseology - the 14-day follow-up window rather than "within 14 days of the due date".
- I find the writing of the results very confusing. This sentence below suggests the outcome was completion rate via telephone versus completion rate via something else. "There was evidence to suggest a difference in completion rate via telephone adjusted OR 0.44 (0.22–0.87, p=0.02) which implies those who received the non-personalised text were more likely to complete the questionnaire when completing via the telephone". I'm wondering why you keep saying via telephone. It is particularly confusing when explaining the results. The last part above again says … those who received the non-personalised text were more likely to complete the questionnaire when completing via the telephone". It looks like the method of completion is the purpose of the study.
- When you use the phrase "were more likely to", you must give the details of the comparison, i.e., more likely than who?
- It is implied, but not adequately explained, that some women completed the questionnaire by some other means. It is not clear how this was handled in terms of the numbers analysed throughout the study and this needs to be explained.
- For your tables 3 and 4, add the number of women. Why does the left column say "response rate for all methods" when the primary outcome is defined as the proportion of the questionnaires completed over the telephone…"
- The heading in the results section, "Response rates for all method", do you mean completion rates for all methods? A response rate is different.
- If you hang your hat on statistically significant evidence, by quoting CIs and p-values, to establish if your SWAT was effective, or not, then the following has no place in your paper. "There is some, non statistically significant, evidence to suggest that there may be a difference in response rate for personalised versus non-personalised text reminders; adjusted OR 0.61 (95% CI 0.30–1.24, p=0.17), in favour of the non-personalised text messages". You cannot say there is non-statistically significant evidence and then support that statement with statistics! Remove this please.
- The heading, "Number of attempts to contact required". Replace with, Number of attempts required to contact the women.
- What do you mean by the maximum number of calls, as stated here "The maximum number of calls was reached for 55 of the 174 participants…"?
- Contacts required is a new term introduced here " There was no evidence of a difference in number of contacts required". What do you mean by it?
- "The average time to respond was 6.2 days (ranging from 5 days early to 103 days late)". Respond to what, the phone call or the text, or the questionnaire?
- "This was similar between those who received a personalised text (8.2 days for early versus 7.1 days for late) and those who received the non-personalised text (4.9 days for early versus 4.7 days for late), but there is a slight difference between those who received personalised or non-personalised texts". If it is similar, how can there be a slight difference? What point are you making here?
- Include the number of participants in the MiQuit Trial earlier in the paper when discussing the 200 randomised for the SWAT.

**Discussion**

- "It did show that there was some evidence that sending a non-personalised text message reminder would have a larger increase in response than sending personalised text messages did". This is misleading because it was not the case when all methods of questionnaire were included. Please amend the statement.
- The final sentence of the discussion needs to be reviewed. What do you mean by overall effectiveness?

Is the work clearly and accurately presented and does it cite the current literature?

Partly

If applicable, is the statistical analysis and its interpretation appropriate?

Partly

Are all the source data underlying the results available to ensure full reproducibility?

Yes

Is the study design appropriate and is the work technically sound?

Yes

Are the conclusions drawn adequately supported by the results?

No

Are sufficient details of methods and analysis provided to allow replication by others?

Partly

Reviewer Expertise:

Epidemiology; Trial Methodology; SWATs

I confirm that I have read this submission and believe that I have an appropriate level of expertise to confirm that it is of an acceptable scientific standard, however I have significant reservations, as outlined above.

# Response

**Published:** 2021-09-09

**Authors:** Elizabeth Coleman

**Affiliations:** University of York, UK, UK

***The authors would like to thank you for a very thorough review of this article - please see responds to your comments and suggestions below. We have amended the article to** **incorporate** **your corrections and suggestions were we pleased it was suitable to do so.***

 Summary

 This was a SWAT within the MiQuit-3 trial. The purpose of the SWAT was to establish if the timing or personalisation of text messages increases completion of a questionnaire. Study design was a 2 x 2 factorial SWAT. Participants who reached their 36-week gestational follow-up were randomised to receive a personalised or non-personalised text message, either one week or one day prior to the telephone follow-up. Primary outcome was completion rate of questionnaire via telephone. Secondary outcomes included: completion rate via any method, time to completion, and number of reminders required. The authors concluded that timing of the text message did not appear to influence the retention of participants. The authors concluded that personalisation of a text message may be detrimental to retention; however, more SWATs should be undertaken in this field.

***Thank you for this well written summary of the paper.***

 Major Comments

 My first comment is on the title, the purpose of the study and the conclusions drawn. The title, correctly refers to the retention of participants in the smoking cessation pregnancy RCT. The purpose of the SWAT though is to evaluate completion of a questionnaire, not retention in the host trial, as claimed in the conclusion in the abstract. I think this is conflated throughout and the authors need to consider this carefully and amend their paper. In fact, the registered SWATs in the NI SWAT repository give the outcomes in both as questionnaire completion. So therefore the conclusions drawn in the abstract and in the discussion are not supported by the data.

***The authors have amended the title of the paper, and changed references to retention within the paper to completion of questionnaires throughout. Alongside making the other suggested changes by the reviewer, we hope to have addressed any concerns in relation to the conclusions drawn.***

 Abstract

 "There was evidence that a personalised text would result in fewer completions via telephone compared with a non-personalised text (adjusted OR 0.44, 95% CI 0.22–0.87, p=0.02)". This statement is confusing. The research question is personalised text message versus non-personalised text message. If using via telephone, then it should be also say non-personalised text via telephone.

***The wording has been amended to attempt to make this statement clearer, and also reference the results for any data collection method.***

 Also, use text message, rather than text, throughout.

***The term ‘text’ has been changed to ‘text message’ throughout the paper.***

 "Personalisation of a text message may be detrimental to retention". I don't think your results support a statement this strong. Firstly, this is not a trial with an adequate sample size to make this claim. Secondly, when you included all methods of receiving the questionnaire (I think this is correct interpretation but it is challenging to establish in the current version of the paper as the detail on all methods is unclear) you did not find that personalised or non-personalised texts mattered. At best you can say, personalisation of a text message appears to affect questionnaire completion via telephone.

***This sentence has been altered to include ‘data was collected only via the telephone’, and now also references that the results do not hold for data collection via all methods. The results are correct as finding of this work, and the conclusion of the abstract references that more work is needed in this area so no other alterations have been made in regards to this comment.***

 Introduction

 I like the succinct introduction.

***Thank you for this comment.***

 Methods

 The methods says "The SWATs are also registered with the Northern Ireland Hub for Trial Methodology Research SWAT Repository (SWATs 35 and 44; both registered December 2015). It's a little confusing to the reader. It looks like you're conducting two SWATs. I understand what you have taken both of these SWATS and conducted the two in a 2 x 2 factorial design. However, make this clear to the reader.

***Additional words have been added to this paragraph to make it clear that this was a factorial SWAT involving these two SWATs.***

 Replace the phrase "carried out" with conducted throughout. I know the phrase carried out is used all the time in literature but it is not correct unless you are describing physically carrying an object.

***The phase carried out has been replaced with conducted.***

 I would use the word women rather than participants, given that it is a trial of pregnant women.

***We thank you for the suggestion – however, the authors have decided not to make this change. The protocol for the MiQuit-3 trial refers to those involved both as participants, and as women. The term participants is not incorrect, and as it is made clear that this work is being done in pregnant women in both the introduction and conclusions of this paper, we feel it does not need to be re-emphasised throughout the whole paper.***

 The phrase "all participants that had not yet had their 36-week gestational follow-up were eligible to participate" suggests that some people were more than 36 weeks pregnant. Is this the case? Otherwise you could simplify it and say women who were 36 weeks pregnant. It's important to be clear on the timing of the intervention given that this is one of your outcomes.

***This sentence references those who were eligible to be included in the SWAT. Some participants in the host trial would have been more than 36 weeks pregnant (or have had their baby/babies) when the SWAT was implement, and thus they were not eligible to be included in the SWAT. The wording of this paragraph has been altered in relation to this comment, and the reviewer comment regarding the number of participants involved in the SWAT, hopefully this has made it clearer.***

 "Participants in MiQuit-3 were blind to their participation in this SWAT". Blinding is different to being unaware. Were they both blinded and unaware?

***A correction has been made in regards to the unawareness and blinding of participants.***

 "The randomisation was undertaken by a statistician independent of the host trial, and of the staff involved in sending the texts". Explain how the statistician did the randomisation, e.g. computer generated. Also, explain how he communicated that randomisation to the researcher assigning the women to each group.

***Details have been added to explain how the randomisation was generated (Stata) and how the randomisation sequence was used.***

 "Block randomisation, stratified by host trial allocation, and whether they had completed the previous follow-up; with varying block sizes of 4, 8, 12 and 16". This is not a sentence.

***Corrections have been made to this sentence.***

 "A £5 voucher was given to all participants who completed a follow-up…". Was this part of the host trial or the SWAT?

***Detail has been added to clarify that this is part of the host trial***

 "…additionally those who provided a saliva sample were given another £30 (£35 total)." Where does the saliva sample come into it? Is this part of the host trial? Explain in the paper.

***Detail has been added to explain why a saliva sample was given, and that it, and associated voucher were part of the host trial.***

 How did you decide on how many to include for the SWAT. I accept that a sample size calculation is not required but a line in the paper on why you decided on (it appears to be 200) would be useful.

***Further details have been added to explain why only 200 were included.***

 In the secondary outcome, explain what you mean by completed by any method.

***Details have been added to explain what other methods of completion could be used.***

 "Time to response, defined as the number of days between the due date of the 36-week gestation follow-up and the date the questionnaire was recorded as complete". Are you certain all follow-up calls were made in 24 hours?

***The date of each attempt to contact was recorded. However this outcome captures completion by any follow-up method.***

 Statistical Analysis

 "The primary outcome was completion rate; defined as the proportion of the questionnaires completed over the telephone within the follow-up window (14 days)". However, you then go on to say that you used logistic regression to analyse this. Logistic regression is not suitable for four categories. I suspect what you mean is that you compared the completion rates across the two personalised/not personalised and again early/late. I can see you did this from the table. However, you need to articulate that in the text because it is currently confusing.

***A logistic model can only deal with two levels of an outcome; it can handle multiple levels of a covariate. The outcome was completion (yes/no), and the swat allocations are included as covariates in the model. Each swat allocation is include as a separate covariate – as stated in the analysis section, providing two, two-level covariates. Thus the model does not include a four level variable, as you have suggested. Had swat allocation been included as a four level covariate, the model would have been able to do this – but it would not have provided an OR for each of the two SWAT interventions separately. This method is the appropriate method for analysing a binary outcome for a factorial SWAT, as such no changes have been made to the text.***

 In statistical analysis, a full stop after level and a new sentence for "As this is a factorial..."

***Thank you for suggesting this correction – it has been made.***

 Full stop required in this sentence too. Also, I suspect the word "compared" in this sentence should read completed. - "Time to response (days between questionnaire due and complete) was analysed using a Cox Proportional Hazards regression, those who compared the questionnaire early had their time set to 0.1, those did not complete were censored at either last contact date or 120 days if not contacted, and those who withdrew in the course of the SWAT were set to their withdrawal date".

***Corrections to this sentence have been made.***

 Again the following is not a sentence, "All models were repeated with the inclusion of an interaction term to explore any possible interactions between the two SWAT interventions; with a significance level of 5%."

***Additional words have been added.***

 Results

 "Additional participants were excluded from the analysis, where the covariates required for the model were not provided". What additional participants? Quantify and explain please. Why were the covariates not 'provided'? Explain please.

***The text in this sentence has been altered to make this clearer.***

 "Three participants were not contacted due to difficulties/adverse events associated with their pregnancy but are still included in the analysis under ITT principles". Commas are required to make sense of the sentence.

***Changes have been made to this sentence.***

 In your flow chart, what does primary refer to, and proximity? Add an explanation or use a term that explains a little better. Also in the flow chart, you say response rate but provide the number of participants. This is not a rate.

***The flow diagram has been updated to include consistent terminology. The numbers in the flow diagram are referencing the numbers within each analysis.***

 In the primary outcome, continue with your phraseology - the 14-day follow-up window rather than "within 14 days of the due date".

***This term has been made consistent throughout the paper.***

 I find the writing of the results very confusing. This sentence below suggests the outcome was completion rate via telephone versus completion rate via something else. "There was evidence to suggest a difference in completion rate via telephone adjusted OR 0.44 (0.22–0.87, p=0.02) which implies those who received the non-personalised text were more likely to complete the questionnaire when completing via the telephone". I'm wondering why you keep saying via telephone. It is particularly confusing when explaining the results. The last part above again says … those who received the non-personalised text were more likely to complete the questionnaire when completing via the telephone". It looks like the method of completion is the purpose of the study.

***The main data collection method for MiQuit-3 was via the telephone. As such, within the SWAT protocol for this factorial SWAT, it was decided that the primary outcome would focus specifically on data collected via this method, and as a secondary explore data collected via any method. The results for the primary and associated secondary outcome have been rewritten slightly to clarify that the results are only including those who completed it via the telephone, as opposed to comparing those who did it via the telephone with something else.***

 When you use the phrase "were more likely to", you must give the details of the comparison, i.e., more likely than who?

***Additional words have been added for clarification.***

 It is implied, but not adequately explained, that some women completed the questionnaire by some other means. It is not clear how this was handled in terms of the numbers analysed throughout the study and this needs to be explained.

***For the secondary outcomes (time to completion, and number of attempts to contact) there have been no additional adjustments or changes to the analysis due to the method of data collection. The completion method is only relevant to the primary outcome, where the trial team detailed that their main method of data collection was via the telephone, and thus the primary outcome explores this. However, to explore the effectiveness of the interventions on any data collection method (as is more typically done in RCTs where a number of methods are utilised) a secondary outcome was to explore this via any method.***

 For your tables 3 and 4, add the number of women. Why does the left column say "response rate for all methods" when the primary outcome is defined as the proportion of the questionnaires completed over the telephone…"

***Response rate, completion rate and retention rate are all frequently used synonymously within the SWAT world. As such the terminology within this paper has switched between the terms. In response to this comment the terminology has been made consistent.***

 The heading in the results section, "Response rates for all method", do you mean completion rates for all methods? A response rate is different.

***Response rate has been changed to completion rate throughout.***

 If you hang your hat on statistically significant evidence, by quoting CIs and p-values, to establish if your SWAT was effective, or not, then the following has no place in your paper. "There is some, non statistically significant, evidence to suggest that there may be a difference in response rate for personalised versus non-personalised text reminders; adjusted OR 0.61 (95% CI 0.30–1.24, p=0.17), in favour of the non-personalised text messages". You cannot say there is non-statistically significant evidence and then support that statement with statistics! Remove this please.

***This section has been reworded to reflect your suggestion.***

 The heading, "Number of attempts to contact required". Replace with, Number of attempts required to contact the women.

***The authors have declined to make this change. In some instances the participant was not contacted, therefore it would be incorrect for the heading to referring the number required to contact them, when some were not contactable. Additionally, as explained in a previous comment we will not be changing participant to women.***

 What do you mean by the maximum number of calls, as stated here "The maximum number of calls was reached for 55 of the 174 participants…"?

***This has been clarified both when describing the outcome, and in the results. The researcher tried to contact the participant a maximum of 6 times.***

 Contacts required is a new term introduced here " There was no evidence of a difference in number of contacts required". What do you mean by it?

***This is referring the number of attempts to contact – a word had been omitted, and has been corrected.***

****

 "The average time to respond was 6.2 days (ranging from 5 days early to 103 days late)". Respond to what, the phone call or the text, or the questionnaire?

***Terminology has been changed.***

 "This was similar between those who received a personalised text (8.2 days for early versus 7.1 days for late) and those who received the non-personalised text (4.9 days for early versus 4.7 days for late), but there is a slight difference between those who received personalised or non-personalised texts". If it is similar, how can there be a slight difference? What point are you making here?

***The wording of the sentence has been changed to clarify this point. The sentence was meant to emphasis the difference in time between the personalised/non-personalised, whilst highlighting the similarities between early/late.***

 Include the number of participants in the MiQuit Trial earlier in the paper when discussing the 200 randomised for the SWAT.

***The authors have included this figure (n=1002) earlier on in the paper.***

 Discussion

 "It did show that there was some evidence that sending a non-personalised text message reminder would have a larger increase in response than sending personalised text messages did". This is misleading because it was not the case when all methods of questionnaire were included. Please amend the statement.

***Additional words have been added to explain that these results are only valid for telephone completion***

 The final sentence of the discussion needs to be reviewed. What do you mean by overall effectiveness?

***These words have been removed.***

# Peer review: Reviewer response for version 1

**DOI:** 10.5256/f1000research.55180.r90364

**Published:** 2021-07-29

**Authors:** Phil J. Edwards

**Affiliations:** 1 Department of Population Health, London School of Hygiene and Tropical Medicine, London, UK

This manuscript reports the results of a 2x2 factorial trial nested within an RCT of MiQuit, a text-message, smoking cessation self-help support programme for pregnant smokers. The nested factorial trial sought to evaluate the effect on response to a questionnaire administered over the telephone of two interventions applied to a pre-notification text message: (i) personalisation (text begins “Hi [name]”, or not), and (ii) timing of text messages (early: one week before follow-up, or late: one day before follow-up).

 194 participants who had not yet had their 36-week gestational follow-up were randomised into this nested trial. Analysis of intervention effects was conducted using a logistic regression model.

 The study found some evidence that personalised text messages reduced response (OR = 0.44; 95% CI 0.22 to 0.87; p=0.02); and no evidence that the earlier timing of text messages had an effect on response (OR = 0.86; 95% CI 0.44 to 1.67; p=0.65).

 The manuscript is a clear and concise account of the study, citing the current literature. The study design is appropriate and the work appears to be technically sound. The authors appropriately recognise that their results are only generalisable to their study population (females aged 17 to 41 years).

 The conclusions are adequately supported by the results, and the study makes a useful contribution to the data collection literature.

Is the work clearly and accurately presented and does it cite the current literature?

Yes

If applicable, is the statistical analysis and its interpretation appropriate?

Yes

Are all the source data underlying the results available to ensure full reproducibility?

Yes

Is the study design appropriate and is the work technically sound?

Yes

Are the conclusions drawn adequately supported by the results?

Yes

Are sufficient details of methods and analysis provided to allow replication by others?

Yes

Reviewer Expertise:

Evidence-based Data Collection

I confirm that I have read this submission and believe that I have an appropriate level of expertise to confirm that it is of an acceptable scientific standard.

# Response

**Published:** 2021-09-09

**Authors:** Elizabeth Coleman

**Affiliations:** University of York, UK, UK

***Thank you for reviewing this article.***
